# Extracellular vesicles as mediators of neuron-glia communication

**DOI:** 10.3389/fncel.2013.00182

**Published:** 2013-10-30

**Authors:** Carsten Frühbeis, Dominik Fröhlich, Wen Ping Kuo, Eva-Maria Krämer-Albers

**Affiliations:** Department of Molecular Cell Biology, Johannes Gutenberg University MainzMainz, Germany

**Keywords:** extracellular vesicles, exosomes, neuron–glia communication, oligodendrocytes, microglia, astrocytes, neuroprotection

## Abstract

In the nervous system, glia cells maintain homeostasis, synthesize myelin, provide metabolic support, and participate in immune defense. The communication between glia and neurons is essential to synchronize these diverse functions with brain activity. Evidence is accumulating that secreted extracellular vesicles (EVs), such as exosomes and shedding microvesicles, are key players in intercellular signaling. The cells of the nervous system secrete EVs, which potentially carry protein and RNA cargo from one cell to another. After delivery, the cargo has the ability to modify the target cell phenotype. Here, we review the recent advances in understanding the role of EV secretion by astrocytes, microglia, and oligodendrocytes in the central nervous system. Current work has demonstrated that oligodendrocytes transfer exosomes to neurons as a result of neurotransmitter signaling suggesting that these vesicles may mediate glial support of neurons.

## INTRODUCTION

Glia cells are involved in central nervous system (CNS) function, development, and maintenance, which all require intense cell–cell communication between glia and neurons. Intercellular communication can be mediated through direct cell–cell contact or paracrine action of secreted molecules. In the past few years, a novel mode of interaction relying on the exchange of extracellular vesicles (EVs) between cells has become evident. Various cell types release EVs of different origin into their environment, which have the potential to transfer a collection of biomolecules between cells locally or over longer distances (Thery, 2011; Raposo and Stoorvogel, 2013). Glia and neurons secrete EVs and the recent literature implicates that intercellular communication by EVs has versatile functional impact in the CNS (Chivet et al., 2012; Frühbeis et al., 2012; Prada et al., 2013; Sharma et al., 2013).

Extracellular vesicles comprise shedding microvesicles (MVs), exosomes, and apoptotic bodies, which differ in size, cargo, membrane composition, and origin. Apoptotic bodies are released during apoptosis, whereas the other types of vesicles are derived from healthy cells (Cocucci et al., 2009; Thery et al., 2009). A mixture of EVs is detectable in virtually all body fluids and to date it is challenging to clearly discriminate the different types, as some classifying criteria are overlapping (Gould and Raposo, 2013). While MVs directly pinch off from the plasma membrane and are heterogeneous in size (up to 1000 nm in diameter), exosomes originate from the endosomal system and exhibit a regular shape (50–100 nm in diameter). Exosomes correspond to the intraluminal vesicles of multivesicular bodies (MVBs), hence their generation involves sorting at the level of the endosomal limiting membrane mediated by the ESCRT (endosomal sorting complex required for transport) machinery (Simons and Raposo, 2009; Baietti et al., 2012) or is assisted by the sphingolipid ceramide and tetraspanins (Trajkovic et al., 2008; Buschow et al., 2009). Fusion of MVBs with the plasma membrane releases exosomes and is controlled by Rab GTPases such as Rab27 in epithelial cells and Rab35 in oligodendrocytes (Hsu et al., 2010; Ostrowski et al., 2010). Exosomes carry characteristic lipids, RNA species, biogenesis-related proteins (Tsg101 and Alix are classic markers), tetraspanins, integrins, heat shock proteins, and cell type specific components. On the other hand, they exclude proteins of other intracellular compartments such as the endoplasmic reticulum and mitochondria (Kalra et al., 2012; Raposo and Stoorvogel, 2013). Less is known about the specific composition and biogenesis of MVs. Interestingly, the molecular machinery for MV generation may necessitate factors also involved in exosome generation (Nabhan et al., 2012).

This review summarizes characteristic properties and functions of EVs emphasizing glial EVs in the CNS and in particular the role of oligodendroglial exosomes in neuron–glia communication.

## BIOLOGICAL FUNCTIONS OF EVs

Since their discovery, several physiological and pathological functions have been ascribed to EVs. Reticulocytes utilize exosome release to eliminate obsolete internal membranes during cell maturation (Harding et al., 2013). Furthermore, RNAs are transported by EVs from cell to cell and can modulate gene expression in the recipient cell. After transfer, mRNAs are translated leading to a new set of proteins in the target cell and miRNAs inhibit the expression of resident proteins (Valadi et al., 2007; Skog et al., 2008; Zhang et al., 2010). In the immune system, antigen presenting cells (APCs) secrete exosomes bearing MHC-peptide complexes, which can activate T-cells, suggesting a role of exosomes in the adaptive immune response. On the other hand, tumor exosomes can induce anti-tumor responses but are also able to facilitate tumor development by suppressing immune responses, stimulating tumor growth, invasion, angiogenesis, and metastasis (Bobrie et al., 2011; Luga et al., 2012; Peinado et al., 2012).

Exosomes also have been implicated in morphogen secretion and thus may mediate evolutionary conserved developmental processes. Exosomes derived from human and *Drosophila* cells carry Wnt in association with its cargo receptor Evi/Wls (Evenness Interrupted/Wntless) on the surface and induce Wnt signaling in target cells (Gross et al., 2012; Gross and Boutros, 2013). At the *Drosophila* larval neuromuscular junction pre-synaptic release of exosomes containing Evi/Wls is required for Wnt transmission to the post-synapse (Korkut et al., 2009; Koles et al., 2012). Moreover, synaptotagmin 4 (Syt4) is transferred via exosomes from pre-synaptic terminals to post-synaptic muscles in turn enabling retrograde Syt4 signaling and synaptic growth (Korkut et al., 2013).

In the mammalian nervous system, cortical neurons release exosomes from somatodendritic compartments. Synaptic glutamatergic activity mediates the rise in post-synaptic calcium levels triggering exosome secretion. As neuronal exosomes carry AMPA receptor subunits, they might play a role in synaptic plasticity by regulating the number of AMPA receptors in the post-synaptic membrane (Lachenal et al., 2011; Chivet et al., 2013). Exosomes thus may be implicated in transsynaptic communication in vertebrates and invertebrates. Intercellular transfer of exosomes may be relevant for pathology in several neurodegenerative diseases, since pathogenic proteins such as prions, β-amyloid peptide, superoxide dismutase, α-synuclein, and tau are released from cells in association with EVs (Bellingham et al., 2012; Schneider and Simons, 2012). These vesicles are assumed to spread the pathogenic proteins throughout the tissue. Moreover, EVs derived from glioma cells carry oncogenic EGFRvIII, RNA, and angiogenic factors. They promote cell transformation and modulate the tumor environment to improve tumor growth (Al-Nedawi et al., 2008; Skog et al., 2008).

## MICROGLIA-DERIVED EVs

Microglia, the resident macrophages of the brain, maintain tissue homeostasis, provide the first line of defense during infection and brain injury, and promote tissue repair. In pathological conditions resting microglia polarize toward a M1 (pro-inflammatory) or M2 (pro-regenerative) phenotype largely defined by the profile of secreted cytokines (Hanisch and Kettenmann, 2007; Saijo and Glass, 2011). Microglia bud MVs of irregular shape and size (0.1–1 μm) from their plasma membrane characterized by high levels of externalized phosphatidylserine. Upon ATP stimulation of P2X_7_ receptors, reactive microglia release MVs carrying the pro-inflammatory cytokine interleukin-1β (IL-1β), the IL-1β-processing enzyme caspase-1, and the P2X_7_ receptor (Bianco et al., 2005). The budding of MVs is facilitated by externalization of acid sphingomyelinase, which induces membrane curvature by locally increasing ceramide levels in the outer leaflet of the plasma membrane (Bianco et al., 2009). The authors suggest that when IL-1β and P2X_7 _containing MVs approach tissue areas with high external ATP levels, MV-associated P2X_7_ receptors become activated, followed by IL-1β processing and release from MVs. This pathway may induce and propagate inflammatory reactions throughout the brain (Prada et al., 2013).

Microglia-derived MVs can transmit inflammatory signals to recipient microglia, which then upregulate the co-stimulatory molecule CD86 and express pro-inflammatory genes like IL-1β, IL-6, inducible nitric oxide synthase, and cyclooxygenase-2 (Verderio et al., 2012). MVs derived from all major types of neural cells and in particular MVs carrying myeloid markers are detectable in the rodent and human CSF under normal conditions. In the inflamed brain, in cases of multiple sclerosis in humans and experimental autoimmune encephalomyelitis (EAE) in mice, the amount of MVs increases dramatically depending on disease severity and the extent of microglia activation. Injection of MVs into the brain of mice with subclinical EAE recruits inflammatory cells to the injection site. However, acid sphingomyelinase deficient mice, which are impaired in MV production, are largely protected from EAE. Intriguingly, FTY720, an oral drug for the treatment of multiple sclerosis, reduces the amount of microglial MVs in the CSF of EAE mice. Microglial MVs thus seem to enforce inflammation in neuroinflammatory diseases such as multiple sclerosis. They may represent promising diagnostic markers or even therapeutic targets of brain inflammation (Colombo et al., 2012).

Intriguingly, microglia-derived MVs can interact with neurons and stimulate spontaneous and evoked excitatory transmission *in vitro* and after injection *in vivo*. Hippocampal neurons exposed to MVs show an increase in miniature excitatory post-synaptic current (mEPSC) frequency without changes in mEPSC amplitude. MVs affect the pre-synaptic site of the excitatory synapse by increasing the release probability of synaptic vesicles through induction of ceramide and sphingosine synthesis. Thus, microglial MVs appear to modulate synaptic activity and enhance neurotransmission (Antonucci et al., 2012; Turola et al., 2012).

Besides MVs, microglia release exosomes with a protein content analogous to B cell- and dendritic cell-derived exosomes (Potolicchio et al., 2005). For example, MHC class II is packed into exosomes and its amount is increasing upon stimulation with interferon-γ. However, whether this is instrumental in antigen presentation and brain immunity is open. In addition, microglial exosomes comprise aminopeptidase N (CD13), which cleaves opioid receptor-binding enkephalins. They also carry enzymes for anaerobic glycolysis and the monocarboxylate transporter 1 (MCT1), potentially delivering energy substrates to target cells, and the insulin degrading enzyme (IDE), which can also degrade the Aβ peptide (Tamboli et al., 2010). It has been suggested that microglial exosome release does not occur constitutively and is induced by Wnt3a, which in turn becomes included in these vesicles (Hooper et al., 2012).

## ASTROCYTE-DERIVED EVs

Astrocytes are multifunctional interactive cells. They are part of the blood brain barrier (BBB), control the extracellular ion balance, provide trophic support, and participate in repair and scarring processes after CNS injury. Similar to microglia, MV shedding from astrocytes is evoked by the ATP-triggered activation of P2X_7_ receptors and subsequent action of acid sphingomyelinase (Bianco et al., 2009). Moreover, astrocytes release vesicles from the cell surface that can be up to 8 μm in size and carry intact mitochondria, and lipid droplets (Falchi et al., 2013). Astrocyte-derived EVs are heterogeneous in their composition and have been ascribed beneficial and pathological functions. MVs and exosomes with proposed physiological functions carry Hsp/Hsc70 and synapsin I implicated in neuroprotection (Taylor et al., 2007; Wang et al., 2011), factors modulating angiogenesis such as FGF-2, VEGF, endostatin, and PEDF (Proia et al., 2008; Hajrasouliha et al., 2013), matrix metallo-proteinases mediating extracellular matrix proteolysis (Sbai et al., 2010), and nucleoside triphosphate diphosphohydrolases (NTDPases; Ceruti et al., 2011). The precise action of these EVs on the level of the target cells, however, remains to be determined.

Astrocyte-derived EVs have been implicated in the propagation of pathogenic proteins in neurodegenerative disorders. Astrocytes expressing mutant SOD1 (copper-zinc superoxide dismutase) secrete increased amounts of exosomes, which carry mutant SOD1. These vesicles can transfer mutant SOD1 to cultured neurons and induce motor neuron death suggesting a role of EVs in the pathogenesis of amyotrophic lateral sclerosis (ALS; Basso et al., 2013). Furthermore, exposure of amyloid peptide to astrocytes triggers the release of pro-apoptotic exosomes that include ceramide and PAR4 (prostate apoptosis response 4). These exosomes are taken up by astrocytes and promote their apoptosis suggesting that exosome-mediated astrocyte death may contribute to neurodegeneration in Alzheimer’s disease (Wang et al., 2012). Exosome-mediated miRNA transfer from astrocytes to neurons has been suggested to participate in neurodegeneration in HIV-associated neurological disorders (Hu et al., 2012). Treatment of cultured astrocytes with pathogenic HIV Tat (trans-activator of transcription) protein and morphine triggers shuttling of miR-29b via exosomes to neuronal cells which results in decreased PDGF-B expression and neuronal viability.

## OLIGODENDROCYTE-DERIVED EXOSOMES AND THEIR ROLE IN AXON-GLIA COMMUNICATION

Oligodendrocytes produce the myelin sheath facilitating impulse conduction. Myelinating oligodendrocytes and axons constitute a sophisticated functional unit based on continuous mutual interaction (Nave, 2010). Hence, maintenance of axonal integrity depends on support from oligodendrocytes. Recent work suggests that this trophic function of oligodendrocytes may relate to the transfer of exosomes from oligodendrocytes to neurons (Frühbeis et al., 2013; Lewis, 2013), in addition to the supply of glycolytic substrates such as lactate (Fünfschilling et al., 2012; Lee et al., 2012). Oligodendrocytes release exosomes that include genuine myelin proteins such as PLP, CNP, MAG, and MOG as well as the NAD-dependent deacetylase sirtuin-2, glycolytic enzymes, and typical exosome-associated proteins such as tetraspanins and heat-shock proteins (Krämer-Albers et al., 2007).

**FIGURE 1:**
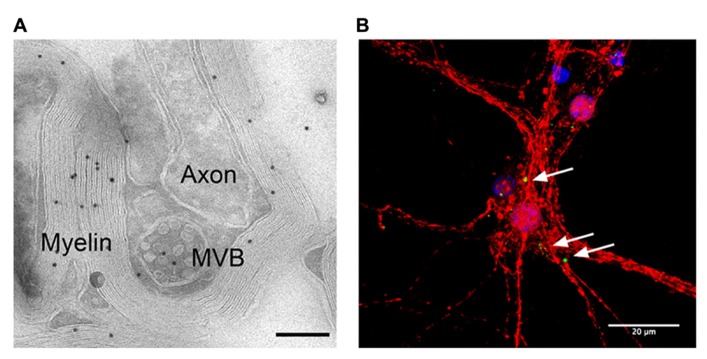
**Adaxonal localization of MVBs and uptake of oligodendroglial exosomes by neurons.** **(A)** Electron micrograph of myelinated axons in the optic nerve labeled with antibodies against PLP (scale bar 200 nm, courtesy of Wiebke Möbius). **(B)** Confocal stack of primary cortical neurons (red) that internalized exosomes, labeled with PLP-EGFP and SIRT2-EYFP (green). Nuclei are blue, scale bar 20 μm.

**FIGURE 2:**
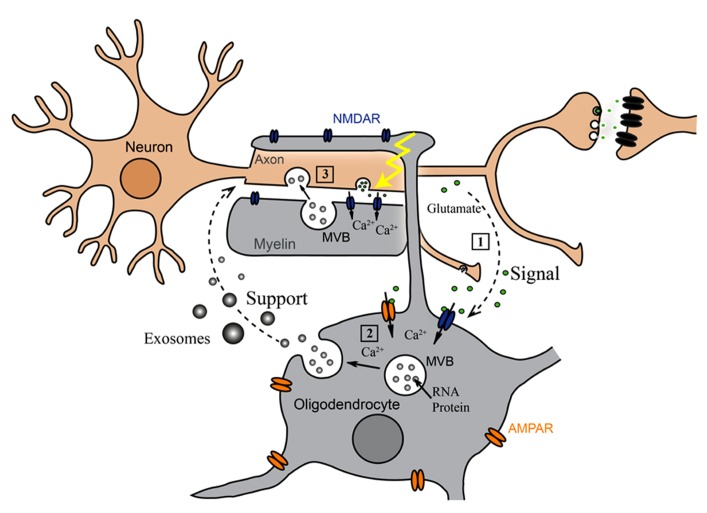
**Oligodendroglial exosomes in neuron-glia communication.** (1) Electrically active axons release glutamate that provokes Ca^2^^+^ entry through oligodendroglial glutamate receptors. (2) Elevation of intracellular Ca^2^^+^ levels triggers exosome release from oligodendrocytes. (3) Neurons internalize exosomes and use their cargo.

Oligodendrocyte-derived exosomes are internalized by a subset of MHC class II negative microglia via macropinocytosis and are subsequently degraded without provoking any response (Fitzner et al., 2011). In addition, they have been suggested to negatively regulate myelin synthesis in an autocrine fashion (Bakhti et al., 2010). However, in myelinated fibers *in situ*, PLP-positive MVBs and their fusion profiles were observed in the innermost non-compacted wrapping of the myelin sheath (**Figure 1A**) implying that exosomes are released into the periaxonal space and involved in axon-glia interaction (Frühbeis et al., 2013). Indeed, the secretion of exosomes from oligodendrocytes is regulated by neurotransmitter signaling. Active neurons release glutamate that provokes Ca^2^^+^ entry through oligodendroglial ionotropic glutamate receptors, mostly of the NMDA subtype. This results in elevation of cytosolic Ca^2^^+^ levels and triggers exosome secretion. In turn, neurons internalize oligodendrocyte-derived exosomes by clathrin and dynamin-dependent endocytosis at both somatodendritic and axonal compartments (**Figure 1B**). The uptake appears selective since astrocytes and oligodendrocytes internalize these exosomes to a minor extent. After internalization, the cargo of oligodendroglial exosomes can be functional in the target neuron. The ectopically expressed enzyme Cre-recombinase is packed into exosomes and activates a reporter in the recipient neuron *in vitro* and, importantly, also after injection of Cre-bearing exosomes into the mouse brain. Moreover, oligodendroglial exosomes improve the metabolic activity of cultured neurons under cell stress. In brief, this suggests a model where active neurons signal to oligodendrocytes and demand the delivery of supportive biomolecules via exosomes (**Figure 2**). Oligodendrocytes can then utilize these vesicles to locally transfer metabolites, protective proteins, glycolytic enzymes, mRNA, and miRNA to axons, which may maintain axonal integrity (Frühbeis et al., 2013). In the peripheral nervous system, evidence suggests that myelinating Schwann cells shuttle supportive cargo to axons by vesicular means facilitating regeneration of axons after injury (Lopez-Verrilli and Court, 2012).

Oligodendroglial trophic support is impaired in CNP and PLP null mice resulting in progressive axonal degeneration (Nave and Trapp, 2008). Since both proteins are components of oligodendroglial exosomes, glial support of axons may be linked to the transfer of substances by exosomes. Lack of these proteins could negatively affect exosome secretion influencing the supply of axons with trophic factors. Indeed, exosome secretion from oligodendrocytes deficient in CNP and PLP is impaired (Frühbeis, Krämer-Albers, unpublished data).

Future work needs to address the identification of surface molecules and target cell receptors mediating vesicle internalization and, moreover, the analysis of functional components which convey support to neurons. Oligodendroglial exosomes include several substances with potentially beneficial activities including stress alleviating proteins and, importantly, mRNA and miRNA. It will be interesting to investigate if RNA transfer via exosomes results in local translation at axonal sites.

## CONCLUSION

Several studies attribute pathological and physiological functions to glial EVs. These vesicles may spread pathogenic factors, promote inflammation, influence neurotransmission, and support neurons. Exosomes secreted by oligodendrocytes transport cargo to neurons and may contribute to axonal integrity. To date, most concepts rely on *in vitro* data and future work will necessitate genetic models to underscore their importance. Nevertheless, EVs emerge as crucial players in the brain and elucidating their physiological relevance opens up new perspectives in CNS research.

## Conflict of Interest Statement

The authors declare that the research was conducted in the absence of any commercial or financial relationships that could be construed as a potential conflict of interest.

## References

[B1] Al-Nedawi K. Meehan B. Micallef J. Lhotak V. May L. Guha A. (2008). Intercellular transfer of the oncogenic receptor EGFRvIII by microvesicles derived from tumour cells. *Nat. Cell Biol.* 10 619–624 10.1038/ncb1725 18425114

[B2] Antonucci F. Turola E. Riganti L. Caleo M. Gabrielli M. Perrotta C. (2012). Microvesicles released from microglia stimulate synaptic activity via enhanced sphingolipid metabolism. *EMBO J.* 31 1231–1240 10.1038/emboj.2011.489 22246184 PMC3297996

[B3] Baietti M. F. Zhang Z. Mortier E. Melchior A. Degeest G. Geeraerts A. (2012). Syndecan-syntenin-ALIX regulates the biogenesis of exosomes. *Nat. Cell Biol.* 14 677–685 10.1038/ncb2502 22660413

[B4] Bakhti M. Winter C. Simons M. (2010). Inhibition of myelin membrane sheath formation by oligodendrocyte-derived exosome-like vesicles. *J. Biol. Chem.* 286 787–796 10.1074/jbc.M110.190009 20978131 PMC3013037

[B5] Basso M. Pozzi S. Tortarolo M. Fiordaliso F. Bisighini C. Pasetto L. (2013). Mutant copper-zinc superoxide dismutase (SOD1) induces protein secretion pathway alterations and exosome release in astrocytes: implications for disease spreading and motor neuron pathology in amyotrophic lateral sclerosis. *J. Biol. Chem.* 288 15699–15711 10.1074/jbc.M112.425066 23592792 PMC3668729

[B6] Bellingham S. A. Guo B. B. Coleman B. M. Hill A. F. (2012). Exosomes: vehicles for the transfer of toxic proteins associated with neurodegenerative diseases? *Front. Physiol.* 3:124 10.3389/fphys.2012.00124 PMC3342525 22563321

[B7] Bianco F. Perrotta C. Novellino L. Francolini M. Riganti L. Menna E. (2009). Acid sphingomyelinase activity triggers microparticle release from glial cells. *EMBO J.* 28 1043–1054 10.1038/emboj.2009.45 19300439 PMC2664656

[B8] Bianco F. Pravettoni E. Colombo A. Schenk U. Moller T. Matteoli M. (2005). Astrocyte-derived ATP induces vesicle shedding and IL-1 beta release from microglia. *J. Immunol.* 174 7268–7277 15905573 10.4049/jimmunol.174.11.7268

[B9] Bobrie A. Colombo M. Raposo G. Thery C. (2011). Exosome secretion: molecular mechanisms and roles in immune responses. *Traffic* 12 1659–1668 10.1111/j.1600-0854.2011.01225.x 21645191

[B10] Buschow S. I. Nolte-`t Hoen E. N. van Niel G. Pols M. S. ten Broeke T. Lauwen M. (2009). MHC II in dendritic cells is targeted to lysosomes or T cell-induced exosomes via distinct multivesicular body pathways. *Traffic* 10 1528–1542 10.1111/j.1600-0854.2009.00963.x 19682328

[B11] Ceruti S. Colombo L. Magni G. Vigano F. Boccazzi M. Deli M. A. (2011). Oxygen-glucose deprivation increases the enzymatic activity and the microvesicle-mediated release of ectonucleotidases in the cells composing the blood-brain barrier. *Neurochem. Int.* 59 259–271 10.1016/j.neuint.2011.05.013 21672581

[B12] Chivet M. Hemming F. Pernet-Gallay K. Fraboulet S. Sadoul R. (2012). Emerging role of neuronal exosomes in the central nervous system. *Front. Physiol.* 3:145 10.3389/fphys.2012.00145 PMC3361079 22654762

[B13] Chivet M. Javalet C. Hemming F. Pernet-Gallay K. Laulagnier K. Fraboulet S. (2013). Exosomes as a novel way of interneuronal communication. *Biochem. Soc. Trans.* 41 241–244 10.1042/BST20120266 23356290

[B14] Cocucci E. Racchetti G. Meldolesi J. (2009). Shedding microvesicles: artefacts no more. *Trends Cell Biol.* 19 43–51 10.1016/j.tcb.2008.11.003 19144520

[B15] Colombo E. Borgiani B. Verderio C. Furlan R. (2012). Microvesicles: novel biomarkers for neurological disorders. *Front. Physiol.* 3:63 10.3389/fphys.2012.00063 PMC3315111 22479250

[B16] Falchi A. M. Sogos V. Saba F. Piras M. Congiu T. Piludu M. (2013). Astrocytes shed large membrane vesicles that contain mitochondria, lipid droplets and ATP. *Histochem. Cell Biol.* 139 221–231 10.1007/s00418-012-1045-x 23108569

[B17] Fitzner D. Schnaars M. Van Rossum D. Krishnamoorthy G. Dibaj P. Bakhti M. (2011). Selective transfer of exosomes from oligodendrocytes to microglia by macropinocytosis. *J. Cell Sci.* 124 447–458 10.1242/jcs.074088 21242314

[B18] Frühbeis C. Fröhlich D Krämer-Albers E. M. (2012). Emerging roles of exosomes in neuron-glia communication. *Front. Physiol.* 3:119 10.3389/fphys.2012.00119 PMC3339323 22557979

[B19] Frühbeis C. Fröhlich D. Kuo W. P. Amphornrat J. Thilemann S. Saab A. S. (2013). Neurotransmitter-triggered transfer of exosomes mediates oligodendrocyte-neuron communication. *PLoS Biol.* 11:e1001604 10.1371/journal.pbio.1001604 PMC3706306 23874151

[B20] Fünfschilling U. Supplie L. M. Mahad D. Boretius S. Saab A. S. Edgar J. (2012). Glycolytic oligodendrocytes maintain myelin and long-term axonal integrity. *Nature* 485 517–521 22622581 10.1038/nature11007 PMC3613737

[B21] Gould S. J. Raposo G. (2013). As we wait: coping with an imperfect nomenclature for extracellular vesicles. *J. Extracell. Vesicles* 2 10.342/jev.v2i0.20389 PMC3760635 24009890

[B22] Gross J. C. Boutros M. (2013). Secretion and extracellular space travel of Wnt proteins. *Curr. Opin. Genet. Dev.* 23 385–390 10.1016/j.gde.2013.02.017 23540564

[B23] Gross J. C. Chaudhary V. Bartscherer K. Boutros M. (2012). Active Wnt proteins are secreted on exosomes. *Nat. Cell Biol.* 14 1036–1045 10.1038/ncb2574 22983114

[B24] Hajrasouliha A. R. Jiang G. Lu Q. Lu H. Kaplan H. J. Zhang H. G. (2013). Exosomes from retinal astrocytes contain anti-angiogenic components that inhibit laser-induced choroidal neovascularization. *J. Biol. Chem.* 288 28058–28067** 10.1074/jbc.M113.470765 23926109 PMC3784718

[B25] Hanisch U. K. Kettenmann H. (2007). Microglia: active sensor and versatile effector cells in the normal and pathologic brain. *Nat. Neurosci.* 10 1387–1394 10.1038/nn1997 17965659

[B26] Harding C. V. Heuser J. E. Stahl P. D. (2013). Exosomes: looking back three decades and into the future. *J. Cell Biol.* 200 367–371 10.1083/jcb.201212113 23420870 PMC3575527

[B27] Hooper C. Sainz-Fuertes R. Lynham S. Hye A. Killick R. Warley A. (2012). Wnt3a induces exosome secretion from primary cultured rat microglia. *BMC Neurosci.* 13:144 10.1186/1471-2202-13-144 PMC3541220 23173708

[B28] Hsu C. Morohashi Y. Yoshimura S. Manrique-Hoyos N. Jung S. Lauterbach M. A. (2010). Regulation of exosome secretion by Rab35 and its GTPase-activating proteins TBC1D10A-C. *J. Cell Biol.* 189 223–232 10.1083/jcb.200911018 20404108 PMC2856897

[B29] Hu G. Yao H. Chaudhuri A. D. Duan M. Yelamanchili S. V. Wen H. (2012). Exosome-mediated shuttling of microRNA-29 regulates HIV Tat and morphine-mediated neuronal dysfunction. *Cell Death Dis.* 3 e381 10.1038/cddis.2012.114 PMC3434655 22932723

[B30] Kalra H. Simpson R. J. Ji H. Aikawa E. Altevogt P. Askenase P. (2012). Vesiclepedia: a compendium for extracellular vesicles with continuous community annotation. *PLoS Biol.* 10:e1001450 10.1371/journal.pbio.1001450 PMC3525526 23271954

[B31] Koles K. Nunnari J. Korkut C. Barria R. Brewer C. Li Y. (2012). Mechanism of evenness interrupted (Evi)-exosome release at synaptic boutons. *J. Biol. Chem.* 287 16820–16834 10.1074/jbc.M112.342667 22437826 PMC3351299

[B32] Korkut C. Ataman B. Ramachandran P. Ashley J. Barria R. Gherbesi N. (2009). Trans-synaptic transmission of vesicular Wnt signals through Evi/Wntless. *Cell* 139 393–404 10.1016/j.cell.2009.07.051 19837038 PMC2785045

[B33] Korkut C. Li Y. Koles K. Brewer C. Ashley J. Yoshihara M. (2013). Regulation of postsynaptic retrograde signaling by presynaptic exosome release. *Neuron* 77 1039–1046 10.1016/j.neuron.2013.01.013 23522040 PMC3626103

[B34] Krämer-Albers E. M. Bretz N. Tenzer S. Winterstein C. Möbius W. Berger H. (2007). Oligodendrocytes secrete exosomes containing major myelin and stress-protective proteins: trophic support for axons? *Proteomics Clin. Appl.* 1 1446–1461 10.1002/prca.200700522 21136642

[B35] Lachenal G. Pernet-Gallay K. Chivet M. Hemming F. J. Belly A. Bodon G. (2011). Release of exosomes from differentiated neurons and its regulation by synaptic glutamatergic activity. *Mol. Cell. Neurosci.* 46 409–418 10.1016/j.mcn.2010.11.004 21111824

[B36] Lee Y. Morrison B. M. Li Y. Lengacher S. Farah M. H. Hoffman P. N. (2012). Oligodendroglia metabolically support axons and contribute to neurodegeneration. *Nature* 487 443–448 10.1038/nature11314 22801498 PMC3408792

[B37] Lewis S. (2013). Glia: transporting cargo from A to B. *Nat. Rev. Neurosci.* 14 589 10.1038/nrn3568 23900413

[B38] Lopez-Verrilli M. A. Court F. A. (2012). Transfer of vesicles from schwann cells to axons: a novel mechanism of communication in the peripheral nervous system. *Front. Physiol.* 3:205 10.3389/fphys.2012.00205 PMC3374349 22707941

[B39] Luga V. Zhang L. Viloria-Petit A. M. Ogunjimi A. A. Inanlou M. R. Chiu E. (2012). Exosomes mediate stromal mobilization of autocrine Wnt-PCP signaling in breast cancer cell migration. *Cell* 151 1542–1556 10.1016/j.cell.2012.11.024 23260141

[B40] Nabhan J. F. Hu R. Oh R. S. Cohen S. N. Lu Q. (2012). Formation and release of arrestin domain-containing protein 1-mediated microvesicles (ARMMs) at plasma membrane by recruitment of TSG101 protein. *Proc. Natl. Acad. Sci. U.S.A.* 109 4146–4151 22315426 10.1073/pnas.1200448109 PMC3306724

[B41] Nave K. A. (2010). Myelination and support of axonal integrity by glia. *Nature* 468 244–252 10.1038/nature09614 21068833

[B42] Nave K. A. Trapp B. D. (2008). Axon-glial signaling and the glial support of axon function. *Annu. Rev. Neurosci.* 31 535–561 10.1146/annurev.neuro.30.051606.094309 18558866

[B43] Ostrowski M. Carmo N. B. Krumeich S. Fanget I. Raposo G. Savina A. (2010). Rab27a and Rab27b control different steps of the exosome secretion pathway. *Nat. Cell Biol.* 12 19–30 10.1038/ncb2000 19966785

[B44] Peinado H. Aleckovic M. Lavotshkin S. Matei I. Costa-Silva B. Moreno-Bueno G. (2012). Melanoma exosomes educate bone marrow progenitor cells toward a pro-metastatic phenotype through MET. *Nat. Med.* 18 883–891 10.1038/nm.2753 22635005 PMC3645291

[B45] Potolicchio I. Carven G. J. Xu X. Stipp C. Riese R. J. Stern L. J. (2005). Proteomic analysis of microglia-derived exosomes: metabolic role of the aminopeptidase CD13 in neuropeptide catabolism. *J. Immunol.* 175 2237–2243 16081791 10.4049/jimmunol.175.4.2237

[B46] Prada I. Furlan R. Matteoli M. Verderio C. (2013). Classical and unconventional pathways of vesicular release in microglia. *Glia* 61 1003–1017 10.1002/glia.22497 23625857

[B47] Proia P. Schiera G. Mineo M. Ingrassia A. M. Santoro G. Savettieri G. (2008). Astrocytes shed extracellular vesicles that contain fibroblast growth factor-2 and vascular endothelial growth factor. *Int. J. Mol. Med.* 21 63–67 18097617

[B48] Raposo G. Stoorvogel W. (2013). Extracellular vesicles: exosomes, microvesicles, and friends. *J. Cell Biol.* 200 373–383 10.1083/jcb.201211138 23420871 PMC3575529

[B49] Saijo K. Glass C. K. (2011). Microglial cell origin and phenotypes in health and disease. *Nat. Rev. Immunol.* 11 775–787 10.1038/nri3086 22025055

[B50] Sbai O. Ould-Yahoui A. Ferhat L. Gueye Y. Bernard A. Charrat E. (2010). Differential vesicular distribution and trafficking of MMP-2, MMP-9, and their inhibitors in astrocytes. *Glia* 58 344–366 19780201 10.1002/glia.20927

[B51] Schneider A. Simons M. (2012). Exosomes: vesicular carriers for intercellular communication in neurodegenerative disorders. *Cell Tissue Res*. 352 33–47 10.1007/s00441-012-1428-2 22610588 PMC3602607

[B52] Sharma P. Schiapparelli L. Cline H. T. (2013). Exosomes function in cell-cell communication during brain circuit development. *Curr. Opin. Neurobiol*. 10.1016/j.conb.2013.08.005 [Epub ahead of print] PMC3830597 23998929

[B53] Simons M. Raposo G. (2009). Exosomes – vesicular carriers for intercellular communication. *Curr. Opin. Cell Biol.* 21 575–581 10.1016/j.ceb.2009.03.007 19442504

[B54] Skog J. Wurdinger T. Van Rijn S. Meijer D. H. Gainche L. Sena-Esteves M. (2008). Glioblastoma microvesicles transport RNA and proteins that promote tumour growth and provide diagnostic biomarkers. *Nat. Cell Biol.* 10 1470–1476 10.1038/ncb1800 19011622 PMC3423894

[B55] Tamboli I. Y. Barth E. Christian L. Siepmann M. Kumar S. Singh S. (2010). Statins promote the degradation of extracellular amyloid \{beta\}-peptide by microglia via stimulation of exosome-associated insulin-degrading enzyme (IDE) secretion. *J. Biol. Chem.* 285 37405–37414 10.1074/jbc.M110.149468 20876579 PMC2988346

[B56] Taylor A. R. Robinson M. B. Gifondorwa D. J. Tytell M. Milligan C. E. (2007). Regulation of heat shock protein 70 release in astrocytes: role of signaling kinases. *Dev. Neurobiol.* 67 1815–1829 10.1002/dneu.20559 17701989

[B57] Thery C. (2011). Exosomes: secreted vesicles and intercellular communications. *F1000 Biol. Rep.* 3 15 10.3410/B3-15 PMC3155154 21876726

[B58] Thery C. Ostrowski M. Segura E. (2009). Membrane vesicles as conveyors of immune responses. *Nat. Rev. Immunol.* 9 581–593 10.1038/nri2567 19498381

[B59] Trajkovic K. Hsu C. Chiantia S. Rajendran L. Wenzel D. Wieland F. (2008). Ceramide triggers budding of exosome vesicles into multivesicular endosomes. *Science* 319 1244–1247 10.1126/science.1153124 18309083

[B60] Turola E. Furlan R. Bianco F. Matteoli M. Verderio C. (2012). Microglial microvesicle secretion and intercellular signaling. *Front. Physiol. * 3:149 10.3389/fphys.2012.00149 PMC3357554 22661954

[B61] Valadi H. Ekstrom K. Bossios A. Sjostrand M. Lee J. J. Lotvall J. O. (2007). Exosome-mediated transfer of mRNAs and microRNAs is a novel mechanism of genetic exchange between cells. *Nat. Cell Biol.* 9 654–659 10.1038/ncb1596 17486113

[B62] Verderio C. Muzio L. Turola E. Bergami A. Novellino L. Ruffini F. (2012). Myeloid microvesicles are a marker and therapeutic target for neuroinflammation. *Ann. Neurol.* 72 610–624 10.1002/ana.23627 23109155

[B63] Wang G. Dinkins M. He Q. Zhu G. Poirier C. Campbell A. (2012). Astrocytes secrete exosomes enriched with proapoptotic ceramide and prostate apoptosis response 4 (PAR-4): potential mechanism of apoptosis induction in Alzheimer disease (AD). *J. Biol. Chem.* 287 21384–21395 10.1074/jbc.M112.340513 22532571 PMC3375560

[B64] Wang S. Cesca F. Loers G. Schweizer M. Buck F. Benfenati F. (2011). Synapsin I is an oligomannose-carrying glycoprotein, acts as an oligomannose-binding lectin, and promotes neurite outgrowth and neuronal survival when released via glia-derived exosomes. *J. Neurosci.* 31 7275–7290 10.1523/JNEUROSCI.6476-10.2011 21593312 PMC6622588

[B65] Zhang Y. Liu D. Chen X. Li J. Li L. Bian Z. (2010). Secreted monocytic miR-150 enhances targeted endothelial cell migration. *Mol. Cell* 39 133–144 10.1016/j.molcel.2010.06.010 20603081

